# EEG Recording and Online Signal Processing on Android: A Multiapp Framework for Brain-Computer Interfaces on Smartphone

**DOI:** 10.1155/2017/3072870

**Published:** 2017-11-16

**Authors:** Sarah Blum, Stefan Debener, Reiner Emkes, Nils Volkening, Sebastian Fudickar, Martin G. Bleichner

**Affiliations:** ^1^Neuropsychology Lab, Department of Psychology, School of Medicine and Health Sciences, University of Oldenburg, Oldenburg, Germany; ^2^Cluster of Excellence Hearing4All, Oldenburg, Germany; ^3^Systems in Medical Engineering Lab, Department of Health Services Research, School of Medicine and Health Sciences, University of Oldenburg, Oldenburg, Germany

## Abstract

**Objective:**

Our aim was the development and validation of a modular signal processing and classification application enabling online electroencephalography (EEG) signal processing on off-the-shelf mobile Android devices. The software application SCALA (Signal ProCessing and CLassification on Android) supports a standardized communication interface to exchange information with external software and hardware.

**Approach:**

In order to implement a closed-loop brain-computer interface (BCI) on the smartphone, we used a multiapp framework, which integrates applications for stimulus presentation, data acquisition, data processing, classification, and delivery of feedback to the user.

**Main Results:**

We have implemented the open source signal processing application SCALA. We present timing test results supporting sufficient temporal precision of audio events. We also validate SCALA with a well-established auditory selective attention paradigm and report above chance level classification results for all participants. Regarding the 24-channel EEG signal quality, evaluation results confirm typical sound onset auditory evoked potentials as well as cognitive event-related potentials that differentiate between correct and incorrect task performance feedback.

**Significance:**

We present a fully smartphone-operated, modular closed-loop BCI system that can be combined with different EEG amplifiers and can easily implement other paradigms.

## 1. Introduction

Electroencephalography (EEG) is a well-established approach enabling the noninvasive recording of human brain-electrical activity. EEG signals refer to voltage fluctuations in the microvolt range and they are frequently acquired to address clinical as well as research questions. Many studies in the research field of cognitive neuroscience rely on EEG, since EEG hardware is available at relatively low cost and EEG signals enable to capture the neural correlates of mental acts such as attention, speech, or memory operations with millisecond precision [1]. Brain-computer interfaces (BCI) typically make use of EEG signals as well [2]. The aim is to identify cognitive states from EEG signatures in real time to exert control without any muscular involvement. BCIs typically benefit from a machine learning signal processing approach [3]. To name a few BCI applications, speller systems provide a communication channel for fully paralyzed individuals (e.g., [4]), motor imagery BCI systems promise controlling prostheses by thought alone [5, 6], and BCI error monitoring systems have been shown to reliably detect car driver emergency braking intentions even before the car driver can hit a brake pedal, thereby supporting future braking assistance systems [7]. A clear drawback of current laboratory BCI technology is that the hardware is often bulky, stationary, and relatively expensive and thereby limits progress.

Furthermore, established laboratory EEG recording technology does not easily allow for the investigating of brain correlates of natural human behaviour. EEG systems, as they are typically used in the lab, include wires connecting scalp electrodes and bulky amplifiers and they do not tolerate human motion during signal acquisition very well [8, 9]. With the recently introduced small, head-mounted wireless EEG amplifiers and their confirmed applicability in real-life situations [10] new paradigms for out-of-the-lab setups are now possible. Head-mounted wireless EEG amplifiers in combination with small notebooks allow for EEG acquisition during natural motion, such as outdoor walking [10] and cycling [11]. Moreover, we recently showed that off-the-shelf Android smartphones can handle stimulus presentation as well as EEG acquisition on a single device [8].

The combination of unobtrusive EEG sensors [8], wireless EEG amplifiers, and smartphone-based signal acquisition and stimulus presentations (which we call transparent EEG [12]) opens up a plethora of possibilities for research, diagnostics, and therapy. The focus on smartphone-operated wearable devices for health and care [13] allows for home-based applications with a high usability. Smartphone are ubiquitous and socially accepted and provide an unparalleled flexibility.

Current smartphone technologies provide sufficient computing power to implement all the steps required for a BCI on a single device, but few groups have attempted to explore this possibility [14].

In previous studies we have shown that Android smartphone-based EEG recordings [10, 15] as well as stimulus presentation on the phone [8] or on a tablet [16] are feasible. However, while the signal quality achieved on handheld devices may be comparable to previous desktop computer-recorded EEG signals [10], all signal processing and classification routines were applied offline on desktop computers, after signal acquisition was concluded. Also, in Debener et al. [8] the temporal precision of auditory events lacked laboratory standard millisecond precision. Debener et al. [8] reported a temporal jitter of approximately 6 ms standard deviation. Stopczynski et al. [9] pioneered an online EEG acquisition and source modelling system running on Android devices. The* Smartphone Brain Scanner* project is freely available and includes real-time visualization of ongoing EEG activity in source space [17]. While confirming the general practicability of on-smartphone processing, the system does not consider delays and processing overheads, as more general processing frameworks would do, and it does not provide a general framework for precise stimulus control and presentation of stimuli, as it is typically required for the implementation of BCI applications. A further drawback is that the* Smartphone Brain Scanner* requires a rooted smartphone and a custom kernel. Another group presented the* NeuroPhone* [18], a BCI application on iPhone. However, while the iPhone application implemented EEG preprocessing and classification along with stimulus presentation and feedback, a laptop was required for EEG signal acquisition. Wang et al. [19] implemented online EEG processing using a frequency coding approach on a mobile device. They reached an impressive classification accuracy (mean = 95.9%) with a steady-state visual evoked potential (SSVEP) paradigm to steer an Android application. In addition to the signal processing, they established EEG data acquisition on the phone but used external hardware for visual stimulus presentation. In a follow-up study, the same work group presented a fully smartphone-operated visual BCI, by integrating stimulus presentation and signal processing on a single mobile device [20]. Their mobile application may be considered the first smartphone-only operated BCI system, but use of proprietary communication protocols and a specific paradigm makes it difficult for others to follow up on this approach.

We present here a fully smartphone-operated, modular closed-loop BCI system. Our system is highly flexible and extendable with regard to the EEG hardware, the experimental paradigm, and the signal processing. Our aim was the development and validation of a reliable, accessible open source software solution for Android smartphone BCIs that allows us to conduct BCI research beyond the lab. A closed-loop BCI system requires time-resolved stimulus presentation, multichannel data acquisition, online data processing and feature extraction, classification, and the delivery of classification outcomes as a feedback signal to the user.

**Figure 1:**
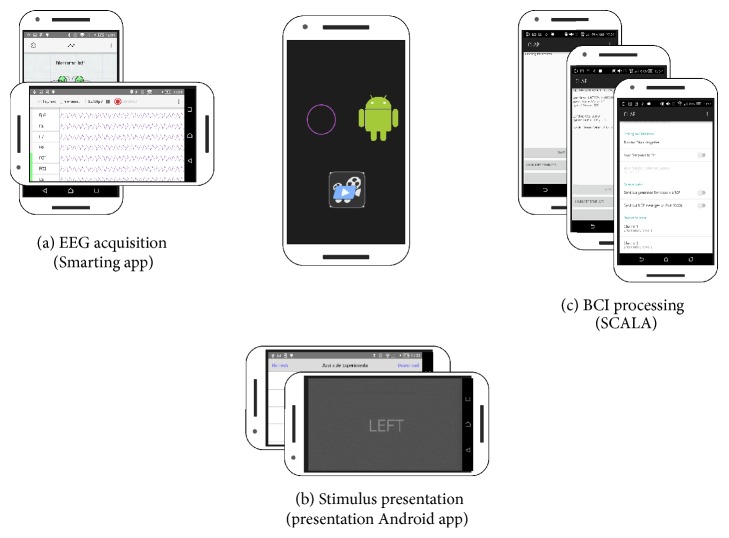
The multiapp BCI on Android approach. An EEG data acquisition application (a) and a stimulus presentation application (b) communicate with our BCI signal processing application SCALA (c). All three applications run on the smartphone during an experiment. They exchange data using socket-based, synchronized communication.

Given our prior experience with smartphone-based EEG acquisition [8], we focused here on integrating available solutions for data recording and stimulus presentation with own signal analysis and classification routines as implemented in a new Android application SCALA (Signal ProCessing and CLassification on Android).  Figure 1 illustrates our multiapp approach where all applications run on the same phone during an experiment. We implemented a highly flexible framework by using well-defined communication protocols and datatypes suitable for different paradigms and different sensor data. We used existing applications for EEG acquisition and stimulus presentation and developed solutions for reliable communication between these applications based on the transmission control protocol (TCP) and the user datagram protocol (UDP). A clear advantage of such a multiapp architecture is that any kind of physiological time series can be processed and that the signal processing application can be easily adapted to different EEG acquisition hardware and different stimulus presentation software solutions.

In the following section we will present our modular system architecture in detail. The timing of the system was evaluated, focusing in particular on auditory event timing in the stimulus presentation application. Finally, the performance of the system was evaluated by employing a reliable selective auditory attention task and comparing our results to previously published reports implementing the identical paradigm offline in the laboratory [22, 23].

## 2. Methods

In this section we describe the software architecture of the signal processing application SCALA and its integration with the EEG acquisition and stimulus presentation applications. Then, we discuss our solution to systematically test the timing of auditory stimulus events on mobile devices. We specify the recording parameters and describe the online and offline signal processing procedures.

### 2.1. The Multiapp Setup

For this study an off-the-shelf Sony Xperia Z1 smartphone (model: C6903; OS: Android 5.1.1) was used for stimulus presentation, data acquisition, and signal processing. Three applications run simultaneously on the same device during an experiment (cf.  Figure 1). Specifically, we used the Smarting Android application for EEG acquisition and storage [24]. The Smarting Android application receives EEG data via Bluetooth from a small, wireless head-mounted 24-channel EEG amplifier and streams signals continuously over the local network via the Labstreaming Layer (LSL) [25]. The EEG samples are time-stamped on the amplifier before they are sent out via Bluetooth which allows for a possible correction of transmission delays on the receiving device. LSL is a framework for the time-stamped acquisition of time series data. The core LSL library is open source and platform-independent. It uses the TCP protocol for a reliable communication between applications in the same network. All applications in our multiapp setup support and include LSL; no additional installation is necessary. For stimulus presentation and experimental control the mobile application from Neurobehavioral Systems' presentation was used (Version 1.0.2 [26]). Presentation performs stimulus presentation with high temporal precision and sends event markers via LSL to the local network. SCALA receives these event markers as well as the EEG data from Smarting and processes them. SCALA in return sends classification results to presentation, which delivers visual feedback to the user.

### 2.2. Software Architecture of SCALA

SCALA has been designed as an Android signal processing application. In order to implement a closed-loop BCI application, it accepts stimulus event marker and time series data streams as inputs (cf.  Figure 1). SCALA can process and classify data streams on a trial-by-trial basis, thereby enabling online signal processing and feedback. The SCALA signal processing pipeline uses a multithreaded setup. Parallel processing in multiple threads was implemented to parallelize data acquisition and signal processing demands. SCALA consists of a general-purpose central-control module and task-specific modules for the signal processing. The general architecture is inspired by the structure of central-control architectures (e.g., Task Control Architecture (TCA) [27]). As a result, SCALA supports task decomposition and time-synchronized processing. In order to achieve maximum hardware flexibility and an easy installation procedure, we used an out-of-the-box Android and omitted the necessity of a customized kernel or root privileges. For user interaction and configuration purposes, SCALA offers a simple graphical user interface (GUI) and the possibility of loading configuration files and data from the phone storage.

**Figure 2:**
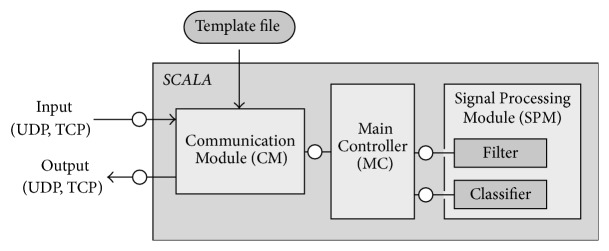
SCALA architecture and functional connections illustrated as a fundamental modelling concepts diagram [21]. Connections with overlaying bullet points indicate bidirectional communication channels. The Communication Module (CM) receives incoming data from several sources and communication protocols. It transmits the data to the Main Controller (MC), which coordinates the signal processing and eventually provides the classification result to the Communication Module. The Signal Processing Module is exchangeable, thereby contributing to the flexibility of SCALA.

A detailed overview of SCALA's system architecture is shown in Figure 2. The* Communication Module (CM)* contains all communication logic. It receives time series data of any kind and discrete event markers using a socket-based communication. The CM continuously receives data from the network but buffers data for processing only when the corresponding event marker to an event of interest is received. The CM stores data in internal data structures and notifies the central controlling module, the* Main Controller (MC).* The MC coordinates the signal processing. It features a bidirectional communication channel to the* Signal Processing Module (SPM)* which contains a filter and a classification submodule. Both submodules are exchangeable and can be adapted to the specific paradigm. Raw data are handed over to the filter and preprocessed data are given back to the MC. The filter type and parameters as well as information about the trial structure can be defined in the settings. The data are preprocessed according to these specifications and are forwarded to the classifier, which extracts one or several features. The classifier in this version of SCALA is a template matching procedure which is described in more detail in the online analysis section. Since SCALA is structured in a modular manner and all communication interfaces are standardized, the signal processing procedure can be different for every paradigm. The classification result is given back to the MC and passed on to the CM. The CM broadcasts the result of the processing pipeline over the local network.

The central coordination of all signal processing steps in the MC has several advantages. Firstly, the single modules do not form any dependencies to external applications or proprietary communication protocols. As a result, SCALA is fully independent of the specific acquisition software and the stimulus presentation software, and therefore, it is independent of the EEG hardware as well. Secondly, the modular architecture facilitates the adaptation to new BCI paradigms and use cases. Further, new Signal Processing Modules can be easily added or replace existing ones. One important module will be an online artefact detection and removal algorithm to deal with nonbrain signals like eye-blinks, muscle artefacts, or heartbeats. Thirdly, any kind of time series data (e.g., EKG or EMG) transmitted as an LSL stream can be received and processed by SCALA. The processing modules are unaware of the type and origin of the data stream since they only receive data from the MC. Only the CM is involved in external and file-based communication. Finally, the CM is the only module with dependencies to Android (GUI, file communication). The other modules can also be used on different platforms and have been tested and validated throughout the development on Linux and Windows systems.

SCALA was developed in Java 1.8 using the Eclipse IDE, release 4.6.1, the Android development tools, and the Android software development kit, revision 25.2.5. SCALA uses a third party open source library [28] for the calculation of a cross-correlation. SCALA is freely available on Github (https://github.com/s4rify/SCALA) under the Apache Commons Free Software License (http://www.apache.org/licenses/LICENSE-2.0).

### 2.3. Timing Test of the Stimulus Presentation

Event-related processing of EEG data requires good temporal precision of event markers. Preferably, markers, for example, indicating the onset of a sound, should be accurate at sampling rate precision. This requirement also holds for online EEG applications such as most BCI paradigms. For the multiapp solution to work well, a reliable communication between the different applications is essential. During the development of SCALA we tested several Android devices and software versions, focusing on the temporal precision between physical stimulus presentation and the recorded event marker. Here we report temporal precision for the hardware/software combination that was finally used for this study (Xperia Z1 smartphone (model: C6903; OS: Android 5.1.1, presentation mobile version 1.0.2)). Since Android is not a real-time operating system, some lag (i.e., a delay between initiating an event and its execution) and jitter (i.e., trial-to-trial variability of the delay) can be expected, in particular in the audio domain. It is known that the audio delay varies between devices and operating system version [29]. By using the EEG acquisition device as an oscilloscope we implemented a simple, easy to replicate, and efficient protocol that allowed us to evaluate and quantify the temporal precision of audio events for different devices and operating systems. The same strategy could be adapted to timing tests in the visual and haptic domain with only minor modifications. The core part of the audio timing test protocol is that the signal on the audio jack is fed directly into the EEG amplifier (to prevent possible damage to the amplifier and a clipped signal, the volume should be set to a medium level) and recorded by the corresponding smartphone app. This setup can measure the time between the programmatic start of the playback of a sound, marked by a stimulus event marker, and the actual playback onset of the sound, as indicated by the audio jack voltage fluctuations, with EEG sampling rate precision (here: 250 Hz sampling rate, resulting in 4 ms precision). This temporal precision is sufficient for most applications.

**Figure 3:**
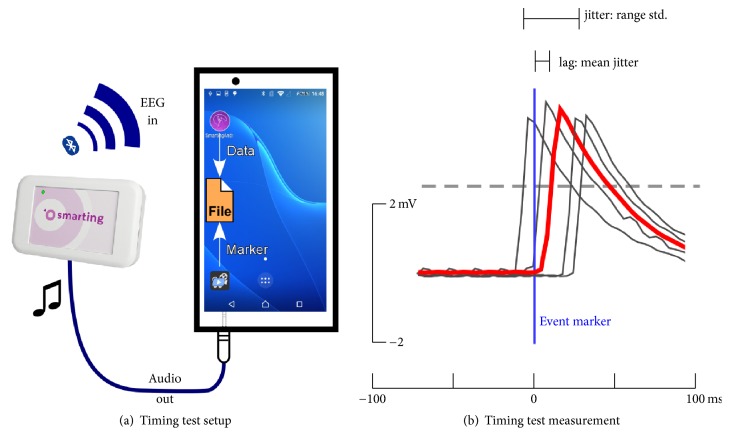
Timing test setup. (a) The varying delay between the programmatic start of a sound playback and the actual onset is evaluated with a smartphone running the Smarting application and the presentation mobile application. A marker sent by presentation indicates the onset of the sound playback and is recorded by the amplifier alongside with the voltage fluctuations fed from the audio jack into the EEG amplifier. The EEG time series is then transmitted wirelessly via Bluetooth to the receiving app on the same smartphone. (b) The difference between the marker (set as reference to 0 ms) and the sound signal (here: the filtered square wave) varies from trial to trial. The single trial latency is defined as the time between marker onset and the amplitude exceeding the half-maximum of the trial averaged response. We define latency jitter as the standard deviation of those single trial latencies. In addition to the jitter properties, the system can also be characterized by its lag, defined as the mean of the single trial latency measures.

The stimulus presentation application plays a sound and sends out an LSL marker indicating the intended playback time, which is recorded into the EEG acquisition file. The sound signal is picked up from the headphone jack and is recorded on a single EEG channel using a cable connection (see Figure 3). Since most audio events have a frequency resolution far above the Nyquist frequency of many EEG amplifiers, we used a square wave audio signal for timing tests. This setup allowed us to quantify the timing of the entire system, while all other experimental details in the stimulus presentation application and the signal processing application were kept constant between timing tests and physiological validation studies. During the timing tests, the EEG amplifier communicates with the Smarting application via Bluetooth, identical to the online usage.

### 2.4. Physiological Validation

**Figure 4:**
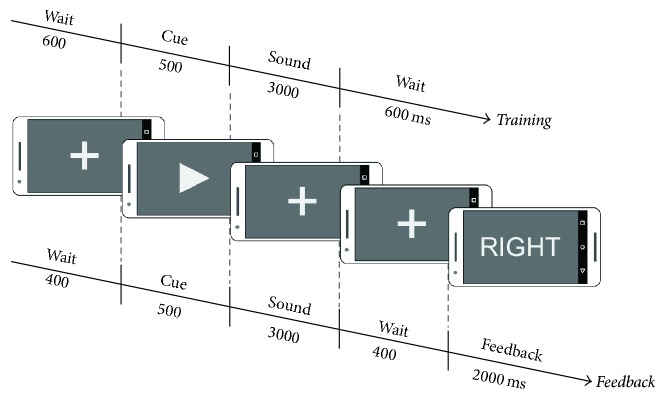
Trial structure of the selective auditory attention paradigm. The upper time axis corresponds to the timing during training trials; the lower time axis corresponds to the timing during feedback trials. Each trial begins with a fixation cross which is shown for either 600 ms during the training or 400 ms during the feedback trials. Then, an arrow tip is presented for 500 ms, pointing to the left or right, indicating the sound direction to be attended. During the sound playback, which lasts 3000 ms, a fixation cross is shown. After the sound playback a break interval of 2400 ms is added. In the feedback trials, the classification outcome is fed back to the user by displaying the word left or right.

We validated the system using a simple auditory attention paradigm that has previously been successfully used to identify selective attention effects on a single trial level. Choi et al. [22] and Bleichner et al. [30] provide a detailed description of this auditory selective attention paradigm. Briefly, three concurrent auditory streams are presented to the subject. Each stream contains a melody, which is composed of single tones. The streams differ in pitch and number of tones (4, 5, and 3 tones) as well as in sound origin (left, right, and centre). Each trial starts with a visual cue, instructing participants to attend either to the left or the right stream; the third, centre stream, is never task relevant (Figure 4). The task is to identify the pitch pattern in the attended stream.

Choi et al. found that auditory attentional modulation is robust enough to be detected on a single trial basis, and this finding was independently replicated in our laboratory [23]. Here we extended the paradigm into an online BCI application, by providing single trial classification outcome feedback to the participants after each trial.

### 2.5. EEG Recording Procedure

Nine participants, which were affiliated to the Neuropsychology Lab Oldenburg, completed the task (6 females; mean age 32 years). The study was approved by the local ethics committee of the University of Oldenburg; informed consent was obtained from all participants. EEG signals were recorded with a wireless amplifier (Smarting, mBrainTrain, Belgrade, Serbia) attached to an electrode cap (EasyCap, Herrsching, Germany). The cap included 24 Ag/AgCl electrodes (international 10/20: Fp1, Fp2, F7, Fz, F8, FC1, FC2, C3, Cz, C4, T7, T8, TP9, TP10, CP5, CP1, CPz, CP2, CP6, P3, Pz, P4, O1, and O2, reference: FCz, ground: AFz). The smartphone was used for recording, stimulus presentation, and online data processing. Recordings were digitized with a sampling rate of 250 Hz and a resolution of 24 bit. Electrode impedances were kept below 10 kΩ. The smartphone was rebooted prior to every session to ensure a minimum of background processes and a maximum of free working memory. Additionally, the phone was kept in Flight Mode to prevent background processes to demand processing time. EEG was recorded in sessions of two blocks with every participant. The first block served as a calibration block to detect the best individual parameters for the online classification. In the second block, consisting of a training and a feedback part, these parameters were then applied online. 40 trials were each presented in the calibration and training block; 120 trials were presented in the feedback block.

### 2.6. Online Analysis

For this paradigm, SCALA recorded EEG data from all 24 channels in the time range of −500 ms to 3500 ms around the stimulus onset. Incoming samples were checked for their timestamps to ensure the corresponding samples for the current trial. Raw data were baseline corrected to the mean of the epoch and bandpass filtered from 1 Hz to 11 Hz. The current filter implementation is a Direct Form II Transposed Filter with coefficients from a 4th-order bandpass Butterworth design. For all further steps in the analysis, only one EEG channel, rereferenced to a mastoid position, was used. Per subject, the most appropriate channel was selected based on the results of a calibration data block prior to the online analysis and the result of a leave-one-out cross validation procedure. Although a multichannel, spatial filter approach should be more effective, a single bipolar channel consisting of a frontocentral electrode and a near mastoid reference site may be sufficiently sensitive to capture auditory evoked potentials [30–32]. Preprocessed channel data were then classified by using a template matching approach [22, 33]. During the training trials, data from all attend-left trials were averaged to form a left-attention template, and data from all attend-right trials were averaged to form a right-attention template. During the feedback trials, a lagged cross-correlation between the current single trial data and both templates was calculated. To compensate for a possible jitter in the stimulus onset, a maximum lag of 32 ms (see timing test results below) was given to the cross-correlation function. The highest correlation indicates the attended side, which is the result of the classification process. The classification procedure used for the online classification was kept deliberately simple as it showed sufficiently good results in prior studies. We refrained from implementing online artefact detection or correction procedures, since our key goal was to evaluate the robustness and quality of the general multiapp framework.

### 2.7. Offline Analysis

Offline analysis of the data was performed using Matlab (Version 2016a, The Mathworks Inc., Natick, MA, United States), EEGLAB Version 13.65b [34] and custom scripts. First, an artefact attenuation procedure based on independent component analysis (ICA) was performed to correct for eye-blinks, eye-movements, and heartbeat artefacts. To this end, the data were 1 Hz high-pass filtered, 60 Hz low-pass filtered (FIR, Hann, −6 dB), and epoched into consecutive segments of 1-second length. Epochs containing nonstereotypical signals were rejected (2 standard deviations criterion, using pop_jointprob) and extended infomax ICA was applied to the remaining data. The resulting ICA weights were applied to the original, unfiltered data and components representing artefacts were automatically detected using the EyeCatch algorithm [35]. The authors of EyeCatch successfully validated their tool against the semiautomatic CORRMAP approach developed in our laboratory [36]. The EyeCatch component selection was confirmed by visual inspection. Finally, artefact attenuation was implemented by back-projection of the remaining, nonartefactual components to the continuous data.

Event-related potentials (ERP) were analysed for the offline artefact-corrected EEG data. We focused on two different events, sound onset responses, which are further referred to as auditory evoked potentials (AEPs), and event-related responses to visual feedback signals. Regarding the former we tested whether an AEP N100 was evident. A poor temporal precision of sound events may result in a small and widespread N100 response with a low signal-to-noise ratio (SNR) and no meaningful topographic distribution. A *t*-test for the vertex (Cz) channel AEPs was used to statistically test whether the N100 amplitude significantly deviated from zero. It is known that negative (i.e., wrong) feedback signals generate the feedback-related negativity (FRN). The FRN is evident as a negative deflection that accompanies feedback indicating negative (compared to favourable) performance outcomes, typically at frontocentral scalp sites [37]. In the present selective attention paradigm, we expected a more negative, FRN-like ERP deflection for incorrect compared to correct classification outcome feedback signals. Note that FRN is typically identified as a difference waveform signal, as it is rarely of absolute negative amplitude, probably due to a larger, overlapping P300-like positive deflection (e.g., [38]).

To determine the offline classification accuracy, a leave-one-out cross validation was implemented. Templates from *n* − 1 trials were calculated and cross-correlated with the left out individual trial. Offline, this was done for each individual EEG channel using as much information for the classification as possible, hence the template of *n* − 1 trials. The classification accuracy per channel is the number of correctly classified trials, divided by the total number of trials. The statistical chance level chance level was calculated after [39].

### 2.8. Post Hoc Online Analysis

Since EEG artefacts can produce spurious classification results, we limited our analysis to an online simulation (from here on: post hoc online) scenario and subsequent offline evaluation. For the post hoc online simulation, we streamed the ICA-cleaned datasets along with corresponding event markers from a computer to the SCALA application on the smartphone. In this online simulation setup, the online processing was identical to the actual online evaluation. The only difference was that the data input stream consisted of artefact-corrected signals.

## 3. Results

### 3.1. System Properties

SCALA performed solidly without crashing once. It found data streams reliably and performed the signal processing fast enough for the given task, and with a deterministic outcome; that is, it always produced the same output for a given initial state or input. SCALA runs on any device running Android (target: Android 6.0, minimum support: Android 4.4.2), as it has no additional hardware requirements (we advise using separate Bluetooth- and Wi-Fi-chips, though). We confirmed the portability of parts of SCALA to different operating systems. Since SCALA's signal processing modules do not have any dependency to the Android OS, they should function properly on any hardware supporting a network communication.

### 3.2. Event Timing Precision

**Figure 5:**
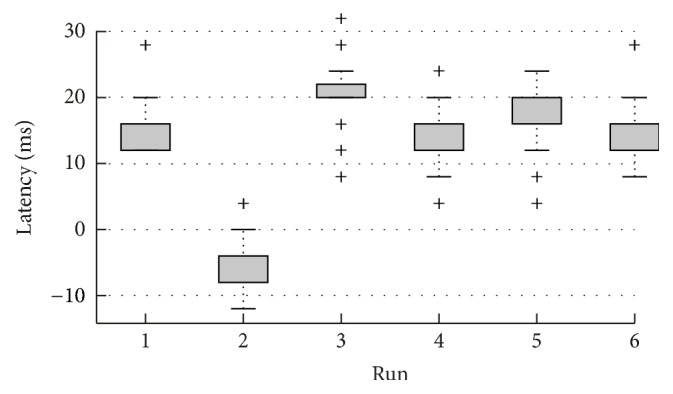
Timing test results for six timing test sessions. Each run consisted of 200 trials, for which the difference between the event marker and the sound onset was recorded (see Figure 3). Each dataset shows the spread of the actual sound onsets after the marker. The tops and bottoms of each box show the 25th and 75th percentiles, respectively; outliers are marked by a cross (>1.5 IQR). Dataset 2 shows the result of one session during which the estimated event markers were placed after the sound onset, leading to a negative deviation from the marker.

The delay and jitter of the auditory playback was measured using the setup depicted in Figure 3. The results of repeated timing tests are shown in Figure 5. The boxplot shows the distribution based on 200 presented stimuli per session in six typical measurement sessions. The data reflect the onset latency, that is, the delay of a sound stimulus event relative to the event marker sent out by the stimulus presentation application. Across all six runs, the average median lag was 12.67 ms (range: −4 to 20 ms). The average within-session jitter was modest, with a mean standard deviation of 2.87 ms (range: 2.31 to 3.23 ms). It is important to notice the difference between within-session and across session event timing precision. Whereas the within-session jitter was rather modest (<3 ms standard deviation), the median lag across sessions varied considerably (average session to session lag difference 14 ms). Moreover, for five out of six datasets the sound started shortly after the marker was sent out, whereas in one dataset the sound onset occurred shortly before the marker was sent out. Since normative data cannot be provided, as they may not generalize to other hardware/software combinations, we refrained from reporting more testing results. The results demonstrate that, whereas the within-session jitter is small and robust, the median lag across sessions may differ significantly.

### 3.3. Event-Related Potentials

**Figure 6:**
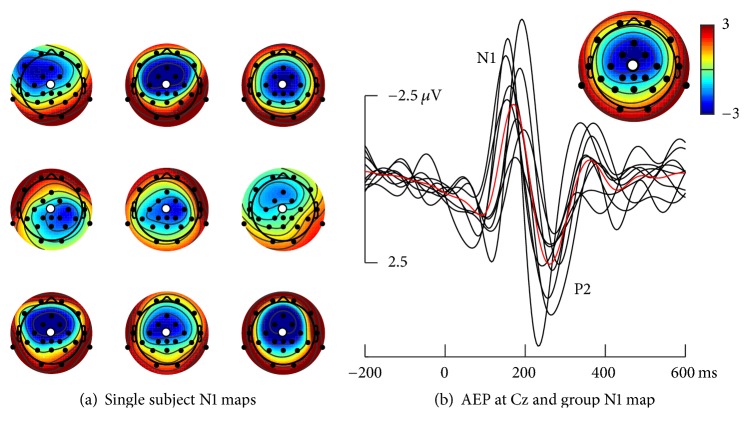
Auditory evoked potentials. (a) Single subject topographic maps, plotted at individual N100 peak latency. Color bar as shown in (b) applies. (b) Single subject (black traces) and group mean AEPs (red trace) at channel Cz, which is indicated as white circle in topographic maps. Topographic map inset shows the group mean N100 topography.

N1 AEP analysis revealed a group mean onset latency of 177 ms (range: 156 to 200 ms), which appears too late for a traditional N100 AEP, which often peaks at around 100 ms (e.g., [32]). However, very similar latencies were reported by Bleichner et al. [30] and Choi et al. [22] who used the identical complex musical sounds. The late N100 latency in this paradigm simply reflects the slowly rising sound onset energy (100 ms cosine squared onset ramp). At electrode Cz, the N100 had a group average amplitude of −2.6 *μ*V (range: −0.6 to −4.6 *μ*V) and differed significantly from zero (*t*(8) = −6.1; *p* < .001). As illustrated in Figure 6, N100 topographies had a frontocentral maximum, with the group mean N100 voltage peaked just anterior to electrode Cz, as could be expected. Moreover, we observed the typical P1-N100-P200 morphology, and N100 amplitude adapted over repeated within-stimulus sound onsets (not shown). Taken together, these results confirm that N100 AEPs could be reliably recorded in this multiapp setup.

**Figure 7:**
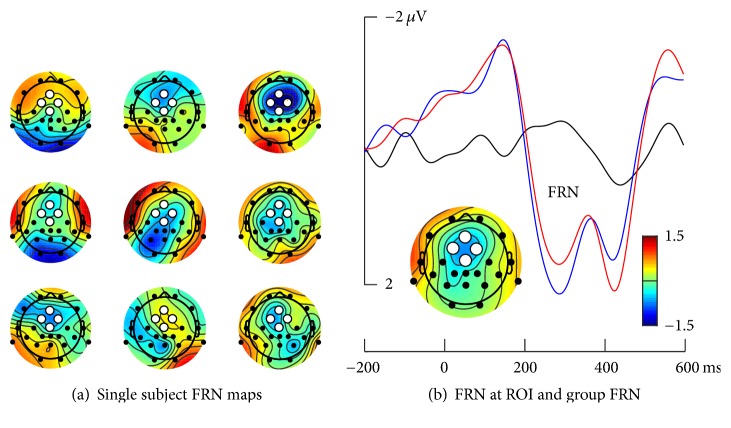
Feedback-related negativity. (a) Single subject topographic maps, plotted as the mean activity in the 250 to 300 ms time interval. Color bar as shown in (b) applies. (b) Group mean FRN at the frontocentral ROI, which is indicated as white circle in the topographic maps in (a). Black trace refers to the difference wave, blue trace to the correct condition, and red trace to the incorrect condition. Topographic map inset shows the group mean FRN topography.

The feedback-related negativity (FRN) was analysed as the mean amplitude in the interval from 250 to 300 ms for a frontocentral region of interest (ROI). The ROI amplitude was obtained by averaging signals from channels Fz, Fc1, Fc2, and Cz. As can be seen in Figure 7, ERPs to those feedback trials where the classification outcome conformed to the cue (correct condition) differed from those where this was not the case (incorrect condition). On average 49.9 trials were available per subject for the correct condition ERPs (range: 27 to 60 trials) and 48.8 trials were available for the incorrect ERPs (range: 21 to 55 trials), and the difference between both was not significant (*t*(8) = 0.69, n.s.). On the other hand, statistical evaluation of the FRN amplitude revealed a significant difference between incorrect and correct conditions, *t*(8) = −2.43 and *p* = .041, in the direction of the predicted effect (Figure 7). As can be seen, the expected frontocentral topography of the FRN was visible in at least five individuals and clearly evident in the group average map. Moreover, the morphology of the difference wave, with a peak at approximately 300 ms conformed to previous FRN reports, which led us to conclude that the FRN was captured in the present dataset.

### 3.4. Classification Performance

**Figure 8:**
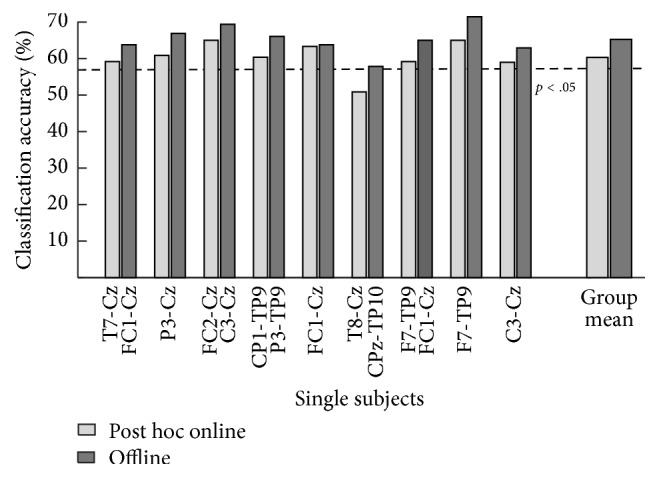
Performance of the classification in the post hoc online analysis and the offline analysis. The empirical chance level of 57.5% for 120 feedback trials is depicted with a dashed line. The subject labels describe the channel pair which was used for the simulated online and the offline analysis. The last two bars show the group mean which show accuracies above chance level for both analysis procedures.

While the online classification on uncorrected data yielded results around the empirical chance level of 57.5% for all participants, the simulated online analysis and the offline analysis on corrected data showed a performance above chance level for all participants but one. The mean decoding accuracy was 60.46% in the post hoc online and 65.51% for the offline analysis. The best subject reached a classification accuracy of 71.43% in the offline analysis; the lowest accuracy was at 50.85% (Figure 8). The different accuracies in the offline and the post hoc online analysis are caused by the different template generation procedures. During the offline analysis, a template of *n* − 1 trials was used to determine the maximal potential of the given channel with as much data as possible. During the post hoc online analysis, a template of 20 trials for each attended side was used to simulate the situation during the online analysis.

## 4. Discussion

Aim of this project was to foster the development of BCI applications for Android smartphones. To this end we developed and evaluated SCALA, a modular BCI software solution for the processing of physiological time series data. We implemented a multiapp approach. This design was chosen because, with the growing needs of multisensor measurements, a modular software approach should provide a higher flexibility than a fully integrated solution. We relied on well-established communication protocols such as LSL. The possibility of streaming any kind of data through LSL offers great flexibility and allows for completely new paradigms, running reliably outside of the lab and on low-cost hardware. We see the decision to develop the signal processing application for an out-of-the-box Android phone as an important step towards ubiquitous computing where computing occurs at any given time at any given place. The development of Android EEG will foster the development of physiological healthcare monitoring applications.

It has been frequently shown that EEG signals can be classified on a trial-by-trial basis, but due to the highly complex nature of the data, and the typically poor SNR, sophisticated machine learning procedures are needed to optimize decoding performance [3, 40]. Here we implemented a rather simple, univariate single channel signal processing pipeline which is conceivable for hearing-aid developments with only a few integrated EEG sensors. The cross-correlation template matching procedure was inspired by Choi et al. [22] and Bleichner et al. [30]. Both groups used a similar approach and reported above chance level classification accuracies. Compared to these studies, which reported a median classification accuracy of approximately 70%, our average classification performance was a little bit lower, yet still above chance level. Several factors may help to explain this modest decline in performance, such as the sample size, the number of trials, modifications in the experimental paradigm, recording conditions, or the hardware. Without a direct comparison approach employing a repeated measurements design it seems impossible to tell whether hardware differences alone are responsible for this decline in performance. In any case, future Android applications would benefit from implementing state-of-the-art machine learning procedures and advanced feature extraction and spatial filter procedures. While this comes with a higher computational demand and thus longer processing time, many BCI paradigms easily tolerate a 100 ms delay or more before feedback is presented. Hence, more complex signal processing appears feasible on Android.

Regarding the ERP signal quality, we observed the typical sound onset AEP N100, replicating our previous smartphone ERP study [8]. Moreover, we found an ERP difference for correct versus incorrect classification feedback. This difference ERP is well known as the FRN and often reported for feedback about negative performance in choice reaction tasks. Others have found that the FRN also follows feedback in a simulated BCI interface [41], with a similar morphology and topography as reported in the present study. Hence we conclude that ERPs can be reliably obtained on smartphone. Moreover, error potentials such as the FRN have clear potential as input features for cognitive or passive BCI paradigms (see [42] for discussion) and can be captured on smartphone as well. A classifier focusing on error-processing brain signals could receive information about its performance during the running session and could adapt its underlying model of classification. Learning classifiers could adapt to the individual user as well as to changing recording environments.

Most ERP studies, and those single trial BCI paradigms making use of time-domain features, benefit from highly accurate event timing. As revealed by our timing test results, our Android setup clearly did not provide perfect precision. However, compared to our previous Android study [8], the latency jitter in the present study was down by at least 50%, to less than 3 ms standard deviation. We speculate that this improvement may be mostly due to the use of a different application solution for stimulus presentation (presentation instead of OpenSesame). Future studies employing direct timing tests using the timing test approach presented here and applied to different stimulus modalities (auditory, visual, and haptic) could reveal whether the presentation app offered by Neurobehavioral Systems and used in the present study indeed provides better event timing than the OpenSesame software environment [43] as used in [8]. Our ERP and BCI results demonstrate that the timing may be sufficient for many applications.

Nevertheless, achieving a better timing on low-cost hardware is still desirable, in particular regarding sporadically occurring outliers, and, more importantly, variations in the mean lag across runs (cf. Figure 5).

Recently, several groups have identified the digital revolution in healthcare delivery (e.g., [44]). Due to the high usability and the large number of integrated sensors, smartphones used as medical devices (e.g., [45]) and as scientific instruments [46, 47] will play a crucial role in the future. While EEG still requires extra hardware, it may soon become part of the increasing family of consumer health wearables [48]. EEG hardware is available for low cost and, in the near future, it may be sufficiently user-friendly and small enough to be taken out of the lab and into real-life situations [8, 12]. In addition, wearable EEG sensor technology seems within reach. In the future, a stable online EEG BCI solution, requiring little more hardware than already available may support several use cases in the growing field of healthcare.

We used the Android platform for our development as it is widely available and allows for flexible app development, despite persisting Android problems in delivering precise audio timing. Our decision for the multiapp architecture required a focus on reliable and widely popular communication standards for achieving between app data flows. This is a worthwhile approach, since the result is a highly flexible solution that can be easily used in combination with other hardware, more advanced signal processing, or different input signals. It should be clearly noted that, in terms of BCI performance, the first closed-loop smartphone BCI application presented by Wang et al. [19, 20] was more powerful than our approach. Yet we provide here an open and flexible platform others can modify to adapt to their needs. Indeed, we regard our solution as a proof-of-concept study, not as a ready-to-go BCI solution for daily life applications. To achieve this long-term goal, further advances in EEG signal processing on the smartphone have to go hand in hand with improvements in the field of EEG hardware and EEG sensor technology (cf. Bleichner & Debener [12] for more details). Smartphone EEG technology has to mature further to play a role in the digital revolution of healthcare that is currently taking place.

In its current state of development, SCALA is an adaptive, stand-alone Android application, which can easily be enhanced by additional modules such as artefact correction. Supplementary modules could then be called consecutively to advance the signal processing. A further approach which would be worthwhile investigating is the use of parts of SCALA as background services, which would not only facilitate the interfacing with different applications, but also save computing time and battery power and thereby facilitate long-term recordings. The authors are currently working on a Java library for artefact detection on Android smartphones. By making the source code freely available to the community (https://github.com/s4rify/SCALA), we hope to foster the development of additions to SCALA to support new use cases in this interesting field of research.

## References

[1] Luck S.

[2] Wolpaw J. R., Birbaumer N., McFarland D. J., Pfurtscheller G., Vaughan T. M. (2002). Brain-computer interfaces for communication and control. *Clinical Neurophysiology*.

[3] Blankertz B., Curio G., Müller K.-R., Dietterich T. G., Becker S., Ghahramani Z. (2002). Classifying single trial EEG: towards brain computer interfacing. *Advances in Neural Information Processing Systems 14 (NIPS 2001)*.

[4] Vansteensel M. J., Pels E. G. M., Bleichner M. G. (2016). Fully implanted brain-computer interface in a locked-in patient with ALS. *The New England Journal of Medicine*.

[5] Braun N., Kranczioch C., Liepert J. (2017). Motor Imagery Impairment in Postacute Stroke Patients. *Neural Plasticity*.

[6] Spataro R., Chella A., Allison B. (2017). Reaching and grasping a glass of water by locked-In ALS patients through a BCI-controlled humanoid robot. *Frontiers in Human Neuroscience*.

[7] Haufe S., Treder M. S., Gugler M. F., Sagebaum M., Curio G., Blankertz B. (2011). EEG potentials predict upcoming emergency brakings during simulated driving. *Journal of Neural Engineering*.

[8] Debener S., Emkes R., De Vos M., Bleichner M. (2015). Unobtrusive ambulatory EEG using a smartphone and flexible printed electrodes around the ear. *Scientific Reports*.

[9] Stopczynski A., Stahlhut C., Petersen M. K. (2014). Smartphones as pocketable labs: Visions for mobile brain imaging and neurofeedback. *International Journal of Psychophysiology*.

[10] Debener S., Minow F., Emkes R., Gandras K., de Vos M. (2012). How about taking a low-cost, small, and wireless EEG for a walk?. *Psychophysiology*.

[11] Zink R., Hunyadi B., Huffel S. V., Vos M. D. (2016). Mobile EEG on the bike: Disentangling attentional and physical contributions to auditory attention tasks. *Journal of Neural Engineering*.

[12] Bleichner M. G., Debener S. (2017). Concealed, Unobtrusive Ear-Centered EEG Acquisition: cEEGrids for Transparent EEG. *Frontiers in Human Neuroscience*.

[13] C. Gretton and M. Honeyman: “The digital revolution: eight technologies that will change health and care | The King’s Fund,” https://www.kingsfund.org.uk/publications/articles/eight-technologies-will-change-health-and-care

[14] Google Research: “TensorFlow: Large-scale machine learning on heterogeneous systems.” pp. 19, 2015

[15] De Vos M., Debener S. (2014). Mobile eeg: towards brain activity monitoring during natural action and cognition. *International Journal of Psychophysiology*.

[16] Griffiths B., Mazaheri A., Debener S., Hanslmayr S. (2016). Brain oscillations track the formation of episodic memories in the real world. *NeuroImage*.

[17] Stopczynski A., Stahlhut C., Larsen J. E., Petersen M. K., Hansen L. K. (2014). The smartphone brain scanner: A portable real-time neuroimaging system. *PLoS ONE*.

[18] Campbell A. T., Choudhury T., Hu S. Neuro-phone: brain-mobile phone interface using a wireless EEG headset.

[19] Wang Y.-T., Wang Y., Jung T.-P. (2011). A cell-phone-based brain-computer interface for communication in daily life. *Journal of Neural Engineering*.

[20] Wang Y.-T., Wang Y., Cheng C.-K., Jung T.-P. Developing stimulus presentation on mobile devices for a truly portable SSVEP-based BCI.

[21] Keller F., Wendt S. FMC: An approach towards architecture-centric system development.

[22] Choi I., Rajaram S., Varghese L. A., Shinn-Cunningham B. G. (2013). Quantifying attentional modulation of auditory-evoked cortical responses from single-trial electroencephalography. *Frontiers in Human Neuroscience*.

[23] Mirkovic B., Bleichner M. G., De Vos M., Debener S. (2016). Target speaker detection with concealed EEG around the ear. *Frontiers in Neuroscience*.

[24] mBrainTrain | Fully Mobile EEG Devices, https://mbraintrain.com/

[25] Swartz Center for Computational Neuroscience and C. Kothe: Lab Streaming Layer (lsl), https://github.com/sccn/labstreaminglayer

[26] C. Neurobehavioral Systems, Inc., Berkeley: Presentation®, https://www.neurobs.com/

[27] Reid S. (1992). Concurrent Planning and Execution for Autonomous Robots. *IEEE Control Systems Magazine*.

[28] The Apache Commons Mathematics Library, Version: 4.0-SNAPSHOT, http://commons.apache.org/proper/commons-math/

[29] Google Developers: Audio Latency Measurements | Android Open Source Project, https://source.android.com/devices/audio/latency_measurements

[30] Bleichner M. G., Mirkovic B., Debener S. (2016). Identifying auditory attention with ear-EEG: CEEGrid versus high-density cap-EEG comparison. *Journal of Neural Engineering*.

[31] Nunez P. L., Srinivasan R. (2006). Electric fields of the brain: the neurophysics of EEG. *Medicine Health Science Books @ Amazon.com*.

[32] Hine J., Debener S. (2007). Late auditory evoked potentials asymmetry revisited. *Clinical Neurophysiology*.

[33] Kerlin J. R., Shahin A. J., Miller L. M. (2010). Attentional gain control of ongoing cortical speech representations in a "cocktail party". *The Journal of Neuroscience*.

[34] Delorme A., Makeig S. (2004). EEGLAB: an open source toolbox for analysis of single-trial EEG dynamics including independent component analysis. *Journal of Neuroscience Methods*.

[35] Bigdely-Shamlo N., Kreutz-Delgado K., Kothe C., Makeig S. EyeCatch: Data-mining over half a million EEG independent components to construct a fully-automated eye-component detector.

[36] Campos Viola F., Thorne J., Edmonds B., Schneider T., Eichele T., Debener S. (2009). Semi-automatic identification of independent components representing EEG artifact. *Clinical Neurophysiology*.

[37] Miltner W. H. R., Braun C. H., Coles M. G. H. (1997). Event-related brain potentials following incorrect feedback in a time-estimation task: evidence for a ‘generic’ neural system for error detection. *Cognitive Neuroscience*.

[38] Huang Y., Yu R. (2014). The feedback-related negativity reflects ‘more or less’ prediction error in appetitive and aversive conditions. *Frontiers in Neuroscience*.

[39] Combrisson E., Jerbi K. (2015). Exceeding chance level by chance: The caveat of theoretical chance levels in brain signal classification and statistical assessment of decoding accuracy. *Journal of Neuroscience Methods*.

[40] Blankertz B., Lemm S., Treder M., Haufe S., Müller K.-R. (2011). Single-trial analysis and classification of ERP components—a tutorial. *NeuroImage*.

[41] Ferrez P. W., Del R. Millán J. (2008). Error-related EEG potentials generated during simulated brain-computer interaction. *IEEE Transactions on Biomedical Engineering*.

[42] Zander T. O., Kothe C. (2011). Towards passive brain-computer interfaces: applying brain-computer interface technology to human-machine systems in general. *Journal of Neural Engineering*.

[43] Mathôt S., Schreij D., Theeuwes J. (2012). OpenSesame: an open-source, graphical experiment builder for the social sciences. *Behavior Research Methods*.

[44] Hollis C., Morriss R., Martin J. (2015). Technological innovations in mental healthcare: Harnessing the digital revolution. *The British Journal of Psychiatry*.

[45] Frist W. H. (2014). Connected health and the rise of the patient-consumer. *Health Affairs*.

[46] Kwok R. (2009). Personal technology: Phoning in data. *Nature*.

[47] Cartwright J. (2016). Smarthphone science: researchers are learning how to convert devices into global laboratories. *Nature*.

[48] Piwek L., Ellis D. A., Andrews S., Joinson A. (2016). The Rise of Consumer Health Wearables: Promises and Barriers. *PLoS Medicine*.

